# Role of Nucleotide-Binding Oligomerization Domain-Containing (NOD) 2 in Host Defense during Pneumococcal Pneumonia

**DOI:** 10.1371/journal.pone.0145138

**Published:** 2015-12-16

**Authors:** Tijmen J. Hommes, Miriam H. van Lieshout, Cornelis van ‘t Veer, Sandrine Florquin, Hester J. Bootsma, Peter W. Hermans, Alex F. de Vos, Tom van der Poll

**Affiliations:** 1 Center for Experimental and Molecular Medicine, Academic Medical Center, University of Amsterdam, Amsterdam, the Netherlands; 2 Center for Infection and Immunity, Academic Medical Center, University of Amsterdam, Amsterdam, the Netherlands; 3 Department of Pathology, Academic Medical Center, University of Amsterdam, Amsterdam, the Netherlands; 4 Laboratory of Pediatric Infectious Diseases, Radboud University Medical Center, Nijmegen, the Netherlands; 5 Division of Infectious Diseases, Academic Medical Center, University of Amsterdam, Amsterdam, the Netherlands; Instituto Butantan, BRAZIL

## Abstract

*Streptococcus (S*.*) pneumoniae* is the most common causative pathogen in community-acquired pneumonia. Nucleotide-binding oligomerization domain-containing (NOD) 2 is a pattern recognition receptor located in the cytosol of myeloid cells that is able to detect peptidoglycan fragments of *S*. *pneumoniae*. We here aimed to investigate the role of NOD2 in the host response during pneumococcal pneumonia. Phagocytosis of *S*. *pneumoniae* was studied in NOD2 deficient (*Nod2* ^*-/-*^) and wild-type (Wt) alveolar macrophages and neutrophils *in vitro*. In subsequent *in vivo* experiments *Nod2* ^*-/-*^ and Wt mice were inoculated with serotype 2 *S*. *pneumoniae* (D39), an isogenic capsule locus deletion mutant (D39Δ*cps*) or serotype 3 *S*. *pneumoniae* (6303) via the airways, and bacterial growth and dissemination and the lung inflammatory response were evaluated. *Nod2* ^*-/-*^ alveolar macrophages and blood neutrophils displayed a reduced capacity to internalize pneumococci *in vitro*. During pneumonia caused by *S*. *pneumoniae* D39 *Nod2* ^*-/-*^ mice were indistinguishable from Wt mice with regard to bacterial loads in lungs and distant organs, lung pathology and neutrophil recruitment. While *Nod2* ^*-/-*^ and Wt mice also had similar bacterial loads after infection with the more virulent *S*. *pneumoniae* 6303 strain, *Nod2* ^*-/-*^ mice displayed a reduced bacterial clearance of the normally avirulent unencapsulated D39Δ*cps* strain. These results suggest that NOD2 does not contribute to host defense during pneumococcal pneumonia and that the pneumococcal capsule impairs recognition of *S*. *pneumoniae* by NOD2.

## Introduction

Invading pathogens are sensed by a wide array of pattern recognition receptors (PRRs) that initiate the innate immune response and shape adaptive immunity [1]. Nucleotide-binding oligomerization domain (NOD)-like receptors (NLRs) are a family of intracellular PRRs that recognize specific microbial components [2]. NOD1 and NOD2 are prominent members of this family. Unlike NOD1, which is expressed by all cell types, NOD2 is mainly expressed by macrophages, monocytes and Paneth cells in the gut [2]. NOD2 recognizes bacterial molecules that are produced during the synthesis and/or degradation of peptidoglycan and is considered to act as a general sensor for most bacteria [3]. NOD2 signals through the adaptor protein receptor interacting protein (RIP)2, ultimately resulting in nuclear factor (NF)-κB translocation to the nucleus and the subsequent production and release of proinflammatory mediators, thereby triggering an immune response aimed at restricting bacterial growth.

*Streptococcus (S*.*) pneumoniae* is the most common causative micro-organism in community-acquired pneumonia and an important cause of mortality world-wide [4,5]. The mortality rate associated with pneumococcal pneumonia ranges from 6 to >40%, largely depending on age and health care settings. Several studies have implicated NOD2 in the recognition of *S*. *pneumoniae* by innate immune cells [6–8]. NOD2 senses internalized pneumococci [6] by a mechanism that depends on lysozyme-dependent digestion of *S*. *pneumoniae* and subsequent delivery of pneumococcal peptidoglycan fragments into the host cell cytosol mediated by pneumolysin, an important virulence factor of this pathogen [8]. During colonization of the upper airways, *S*. *pneumoniae* recognition by NOD2 induces the production of CC-chemokine ligand 2, leading to the recruitment of inflammatory macrophages necessary for bacterial clearance [8].

Thus far the role of NOD2 in lower respiratory tract infection by *S*. *pneumoniae* has not been studied. Here we set out to determine the contribution of NOD2 to the host response during pneumococcal pneumonia by infecting NOD2 deficient (*Nod2* ^*-/-*^) mice with a variety of wild-type (Wt) and genetically modified *S*. *pneumoniae* strains via the airways.

## Materials and Methods

### Ethics statement

Experiments were carried out in accordance with the Dutch Experiment on Animals Act and approved by the Animal Care and Use Committee of the University of Amsterdam (Permit number DIX100121).

### Mice

*Nod2* ^*-/-*^ C57BL/6 mice were purchased from Jackson Laboratories (Bar Harbor, ME). Age and sex matched Wt C57BL/6 mice were purchased from Charles River (Maastricht, the Netherlands) and maintained at the animal care facility of the Academic Medical Centre (University of Amsterdam), according to national guidelines with free access to food and water.

### Phagocytosis

Phagocytosis of *S*. *pneumoniae* by alveolar macrophages and blood neutrophils from *Nod2* ^*-/-*^ and Wt mice was done in essence as previously described [9]. In brief, *S*. *pneumoniae* (serotype 2, D39) were cultured as described below and washed with pyrogen-free sterile saline and resuspended in sterile PBS to a concentration of 2×10^9^ bacteria/ml. The concentrated *S*. *pneumoniae* preparation was treated for 1 h at 37°C with 50 μg/ml Mitomycin C (Sigma-Aldrich; Zwijndrecht, the Netherlands) to prepare alive but growth-arrested bacteria. Subsequently, the growth-arrested *S*. *pneumoniae* preparation was washed twice in ice-cold sterile PBS by centrifugation at 4°C, and the final pellet was dispersed in ice-cold PBS in the initial volume and transferred to sterile tubes. Undiluted samples of these preparations failed to generate any bacterial colonies when plated on blood agar plates, indicating successful growth arrest. Growth-arrested bacteria were labeled with carboxyfluorescein succinimidyl ester (CFSE, Invitrogen, Breda, the Netherlands). 100 μl heparinized whole blood from Wt and *Nod2* ^*-/-*^ mice was incubated with 10 μl bacteria in RPMI (end concentration of 1 x 10^7^ bacteria/ml) at 37°C (n = 8 per group) or 4°C (n = 4 per group). After 60 minutes, samples were put on ice to stop phagocytosis. Afterwards, red blood cells were lysed using isotonic NH_4_Cl solution (155 mM NH_4_Cl, 10 mM KHCO_3_, 100 mM EDTA, pH 7.4). Neutrophils were labeled using anti-Ly-6G-PE (clone 1A8, BD Pharmingen, San Diego, CA) and washed twice in FACS-buffer (0.5% BSA, 0.01% NaN_3_, 0.35 mM EDTA in PBS) for analysis. Subsequently, alveolar macrophages from 8 individual Wt and *Nod2* ^*-/-*^ mice were obtained as described elsewhere [10]. Briefly, the trachea was exposed through a midline incision and cannulated with a sterile 22-gauge Abbocath-T catheter (Abbott). Bronchoalveolar lavage was performed by instilling three 0.5 ml aliquots of sterile saline. Total cell numbers were counted from each sample using a hemacytometer. Cells were washed twice and resuspended in RPMI containing 2 mM L-glutamine and 10% FCS. 1 x 10^5^ cells per well were seeded in a 96-well flat-bottom plate in 100 μL to adhere overnight at 37°C, 5% CO_2_. The following day, macrophages were washed with pre-warmed medium to wash away non-adherent cells. Growth-arrested bacteria were opsonized for 30 minutes at 37°C in 10% normal mouse serum and washed twice in PBS before they were added to the cells at a multiplicity of infection of 100 in a volume of 10 μL. Bacteria and macrophages were spun at 1000 RPM for 5 minutes and incubated at 37°C (n = 8 wells per strain) or 4°C (n = 4 wells per strain). After 1 hour, samples were washed with ice-cold PBS, then thoroughly scraped from the bottom and washed again in FACS-buffer. The degree of phagocytosis was determined using FACSCalibur (Becton Dickinson Immunocytometry, San Jose, CA) The phagocytosis index of each sample was calculated as follows: geometric mean fluorescence x % positive cells.

### Induction of pneumonia

Pneumococcal pneumonia was induced by either serotype 2 *S*. *pneumoniae* (D39), a non-encapsulated mutant strain (isogenic capsule locus(cps) deletion mutant D39Δ*cps*) of D39 [11,12] or a serotype 3 pneumococcal strain (ATCC 6303, American Type Culture Collection, Manassas, VA). All bacterial strains were grown for 3–6 hours to midlogarithmic phase at 37°C in Todd-Hewitt broth (Difco, Detroit, MI), supplemented with yeast extract (0.5%). Bacteria were harvested by centrifugation at 4000 rpm, and washed twice in sterile isotonic saline. Next, mice (n = 8 per strain for each time point), were inoculated with 10^7^ colony forming units (CFU) D39, 10^8^ CFU D39Δ*cps* or 5 x 10^4^ CFU serotype 3 *S*. *pneumoniae* per mouse in a 50 μl saline solution and sacrificed 6, 24 or 48 hours thereafter as described [9,12]. All mice survived to the predefined endpoint in all experiments. Collection and handling of samples were done as described [9,12]. In brief, blood was drawn into heparinized tubes and organs were removed aseptically and homogenised in 4 volumes of sterile isotonic saline using a tissue homogenizer (Biospec Products, Bartlesville, UK). To determine bacterial loads, ten-fold dilutions were plated on blood agar plates and incubated at 37°C for 16 hours.

### Assays

Lung homogenates were prepared for immune assays as described [9]. Tumor necrosis factor (TNF)-α, interleukin (IL)-1-β, IL-6, Keratinocyte-derived chemokine (KC), macrophage inflammatory protein 2 (MIP-2), chemokine (C-C motif) ligand 2 (CCL2) (all R&D systems, Minneapolis, MN) and myeloperoxidase (MPO; Hycult Biotechnology BV, Uden, the Netherlands) were measured using specific ELISAs according to manufacturers’ recommendations.

### Histology

After embedding, lungs were stained with haematoxylin and eosin. To score lung inflammation and damage, the entire lung surface was analyzed with respect to the following parameters: bronchitis, edema, interstitial inflammation, intra-alveolar inflammation, pleuritis, endothelialitis and percentage of the lung surface demonstrating confluent inflammatory infiltrate. Each parameter was graded 0–4, with 0 being ‘absent’ and 4 being ‘severe’. The total pathology score was expressed as the sum of the score for all parameters. Granulocyte staining was done using FITC-labeled rat anti-mouse Ly-6G mAb (Pharmingen, San Diego, CA, USA) as described earlier [9]. Ly-6G expression in the lung tissue sections was quantified by digital image analysis [13]. In short, lung sections were scanned using the Olympus Slide system (Olympus, Tokyo, Japan) and TIF images, spanning the full tissue section were generated. In these images Ly-6G positivity and total surface area were measured using Image J (U.S. National Institutes of Health, Bethesda, MD, http://rsb.info.nih.gov/ij); the amount of Ly-6G positivity was expressed as a percentage of the total surface area.

### Statistical analysis

Data are expressed as box-and-whisker diagrams depicting the smallest observation, lower quartile, median, upper quartile and largest observation, or as medians with interquartile ranges as indicated. Comparisons between groups were conducted using the Mann-Whitney *U*. All analyses were done using GraphPad Prism version 5.01 (GraphPad Software, San Diego, CA). *P*-values less than 0.05 were considered statistically significant.

## Results

### NOD2 deficiency reduces the capacity of alveolar macrophages and neutrophils to phagocytose *S*. *pneumoniae in vitro*

**Fig 1:**
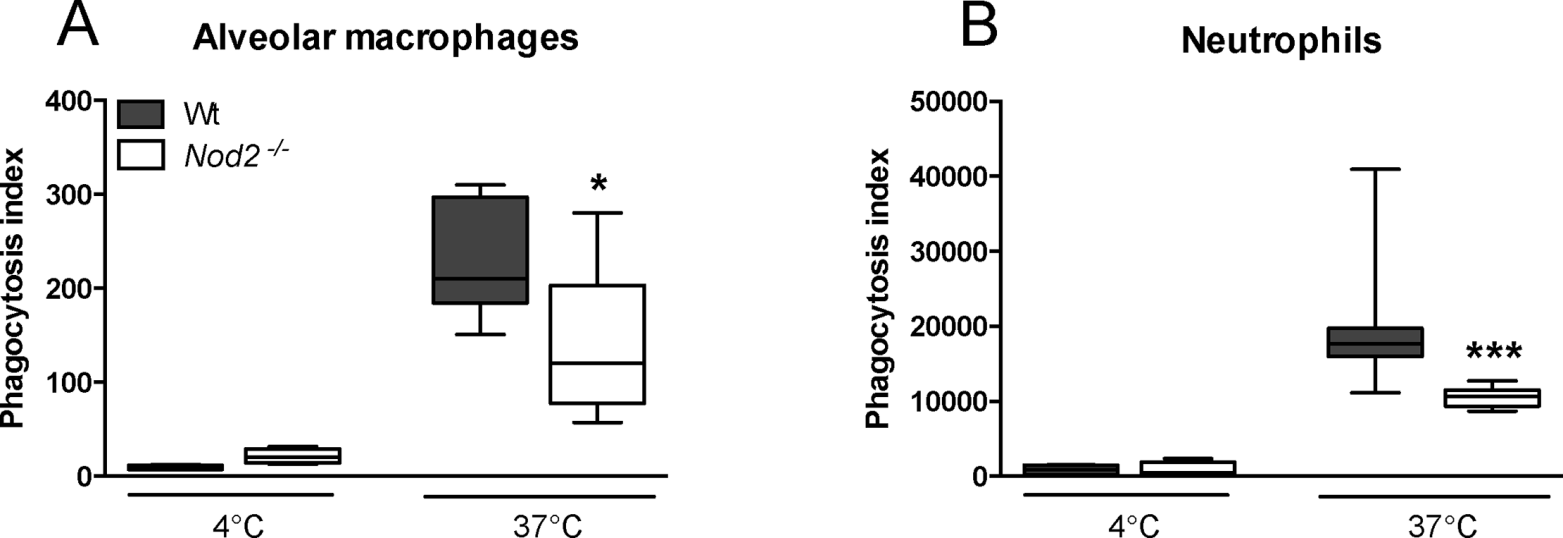
NOD2 deficiency reduces the capacity of alveolar macrophages and neutrophils to internalize *S*. *pneumoniae in vitro*. Growth arrested, FITC labeled *S*. *pneumoniae* D39 were incubated with alveolar macrophages (A) or CFSE-labeled *S*. *pneumoniae* D39 with peripheral blood neutrophils (B) from wild-type (Wt) and *Nod2* ^*−/−*^ mice at 4°C (n = 3–4 per mouse strain) or 37°C (n = 6–8 per mouse strain) for 1 hour after which phagocytosis was quantified. Data are expressed as box-and-whisker diagrams depicting the smallest observation, lower quartile, median, upper quartile and largest observation; *P<0.05, ***P<0.001 versus Wt cells.

Since NOD2 has been shown to promote phagocytosis of *Staphylococcus aureus* [14], we first tested the capacity of *Nod2* ^*-/-*^ and Wt leukocytes to internalize *S*. *pneumoniae* (D39) *in vitro*. To this end we harvested alveolar macrophages and whole blood from naïve *Nod2* ^*-/-*^ and Wt mice and quantified fluorochrome labelled internalized *S*. *pneumoniae* by FACS. Interestingly, both *Nod2* ^*-/-*^ alveolar macrophages and blood neutrophils showed impaired internalization of *S*. *pneumoniae in vitro* (Fig 1A and 1B; *P*<0.05 and *P*<0.001 respectively).

### NOD2 deficiency does not impact on bacterial growth or dissemination during pneumonia caused by serotype 2 *S*. *pneumoniae* (D39)

**Fig 2:**
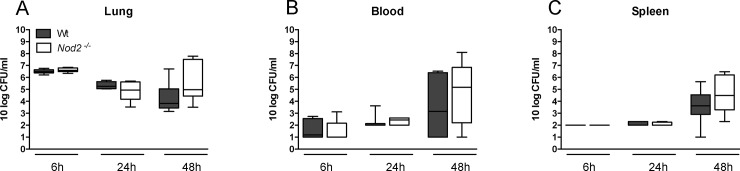
NOD2 deficiency does not impact on bacterial growth or dissemination during pneumonia caused by serotype 2 *S*. *pneumoniae* (D39). Wild-type (Wt) and *Nod2* ^*−/−*^ mice were intranasally infected with 10^7^ CFU of *S*. *pneumoniae* and sacrificed 6, 24 or 48 hours later. Bacterial loads were determined in lung homogenates (A), blood (B) and spleen (C). Data are expressed as box-and-whisker diagrams depicting the smallest observation, lower quartile, median, upper quartile and largest observation (8 mice per group at each time point). Differences between groups were not significant.

To test whether the reduced phagocytic capacity of leukocytes *in vitro* results in impaired host defense during pneumonia *in vivo*, we infected *Nod2* ^*-/-*^ and Wt mice with *S*. *pneumoniae* D39 via the airways. Next we harvested blood and organs 6, 24 and 48 hours later to determine bacterial burdens. We observed no differences in bacterial loads in lungs, blood or spleens between *Nod2* ^*-/-*^ and Wt mice (Fig 2).

### NOD2 deficiency does not impact on inflammation during pneumonia caused by serotype 2 *S*. *pneumoniae* (D39)

**Fig 3:**
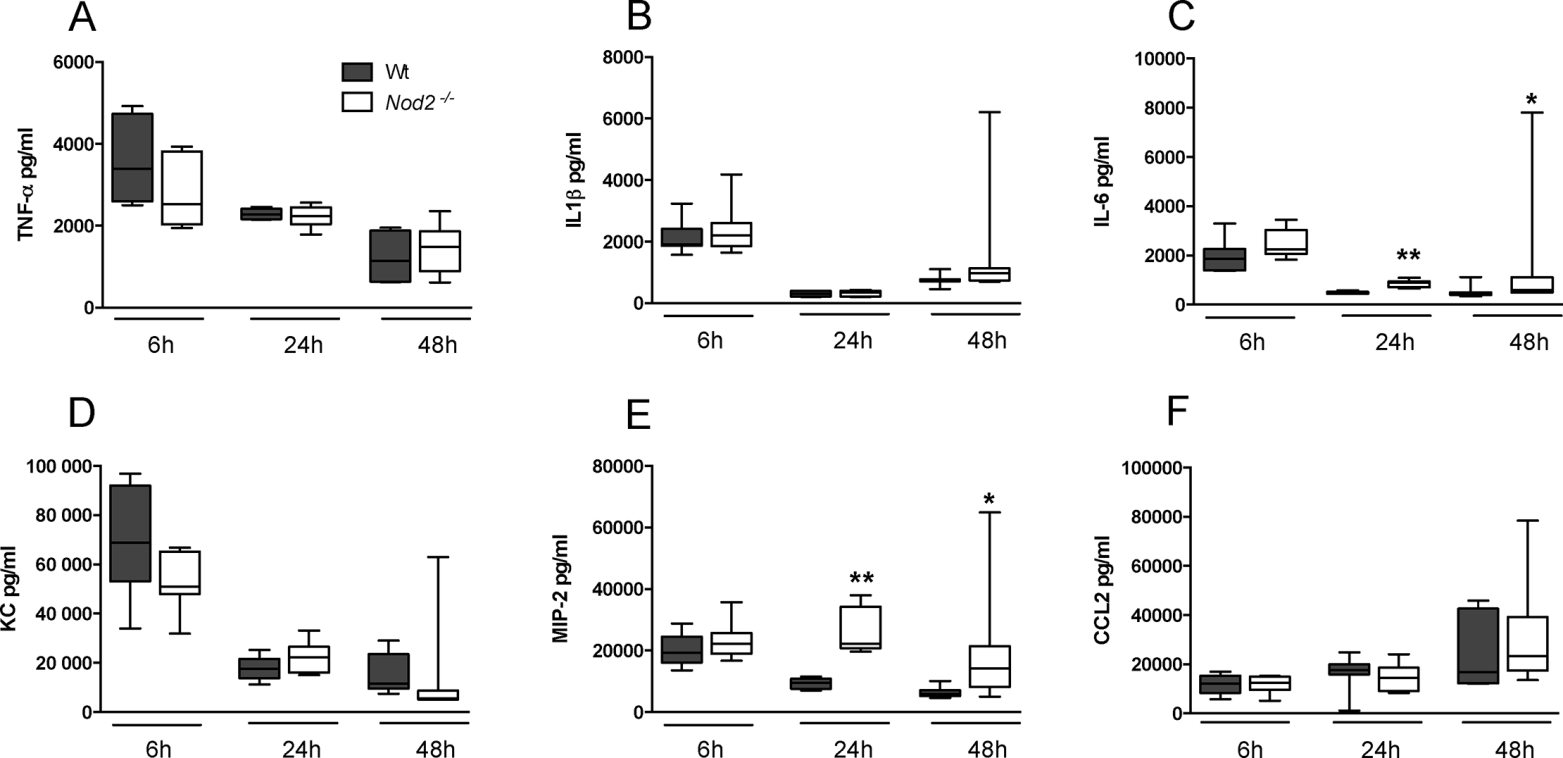
Cytokine and chemokine concentrations in lung homogenates of wild-type and *Nod2* ^*-/-*^mice during pneumococcal pneumonia caused by serotype 2 *S*. *pneumoniae* (D39). Proinflammatory cytokine (TNF-α, IL-1β, IL-6) and chemokine (KC, MIP-2 and CCL2) levels in lung homogenates at 6, 24 and 48 hours after intranasal *S*. *pneumoniae* D39 infection in wild-type (Wt) and *Nod2* ^*-/-*^ mice. Data are expressed as box-and-whisker diagrams depicting the smallest observation, lower quartile, median, upper quartile and largest observation (8 mice per group at each time point); * *P*<0.05, ** *P*<0.01.

**Fig 4:**
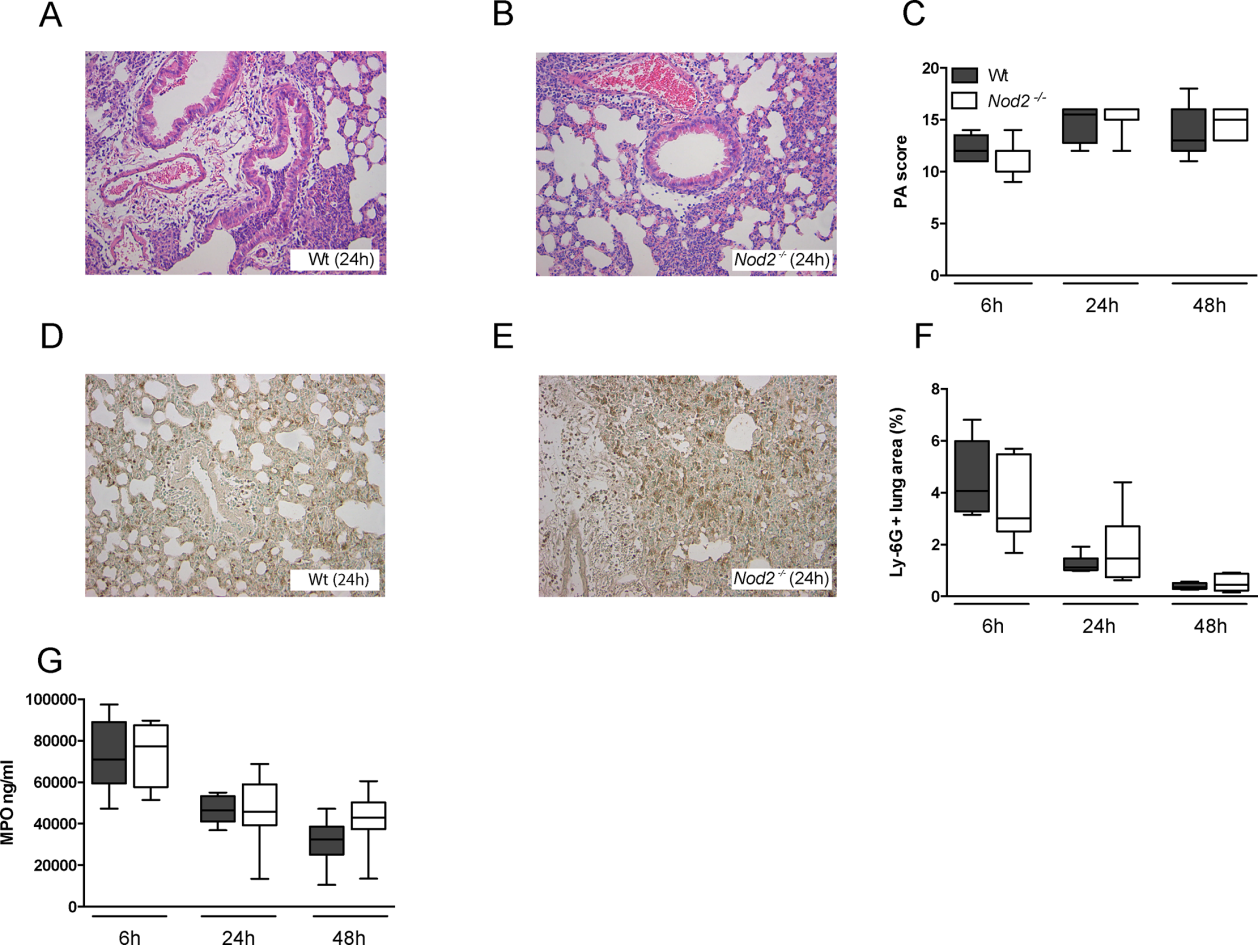
NOD2 deficiency does not influence lung pathology and neutrophil recruitment during pneumonia caused by serotype 2 *S*. *pneumoniae* (D39). Wild-type (Wt) and *Nod2* ^*−/−*^ mice were intranasally infected with 10^7^ CFU of *S*. *pneumoniae* D39 and sacrificed 6, 24 or 48 hours later. Representative hematoxylin and eosin (HE) stainings of lung tissue of Wt (A) and *Nod2* ^*-/-*^ mice (B) 24 hours after inoculation with *S*. *pneumoniae* D39 (original magnification ×200). Quantification of pulmonary Ly-6G positivity (F) and MPO levels in whole lung homogenates (G) 6, 24 or 48 hours after intranasal infection with *S*. *pneumoniae* D39 of wild-type (Wt) and *Nod2* ^*−/−*^ mice. Representative neutrophil stainings (brown) of Wt (D) and *Nod2* ^*−/−*^ mice (E) 24 hours after induction of pneumococcal pneumonia are shown (original magnification ×200). Data are expressed as box-and-whisker diagrams depicting the smallest observation, lower quartile, median, upper quartile and largest observation (8 mice per group at each time point). Differences between groups were not significant.

Considering that NOD2 has been found to contribute to *S*. *pneumoniae* induced inflammatory responses in macrophages [6], we studied the extent of lung inflammation in *Nod2* ^*-/-*^ and Wt mice during pneumonia. To this end we measured cytokines and chemokines in whole lung homogenates obtained from *Nod2* ^*-/-*^and Wt mice at several time points after infection. We found elevated IL-6 levels in lung homogenates taken from *Nod2* ^*-/-*^ mice at 24 and 48 hours post-infection (Fig 3C; *P*<0.05 and *P*<0.01). Similarly at 24 and 48 hours post-infection *Nod2* ^*-/-*^ displayed increased levels of MIP-2 in their lungs compared to Wt mice (Fig 3E; *P*<0.01 and *P*<0.05). Lung pathology, semi-quantitatively scored by methods previously described [9] (Fig 4A–4C), was not different between strains. Neither was the extent of neutrophil recruitment into the lungs, measured by the number of Ly6G-positive cells in lung tissue (Fig 4D–4F) and MPO concentrations in whole lung homogenates (Fig 4G). These data argue against a role for NOD2 in lung inflammation during pneumonia caused by *S*. *pneumoniae* D39.

### NOD2 deficiency results in defective pulmonary clearance of non-encapsulated serotype 2 *S*. *pneumoniae* (D39Δ*cps*)

**Fig 5:**
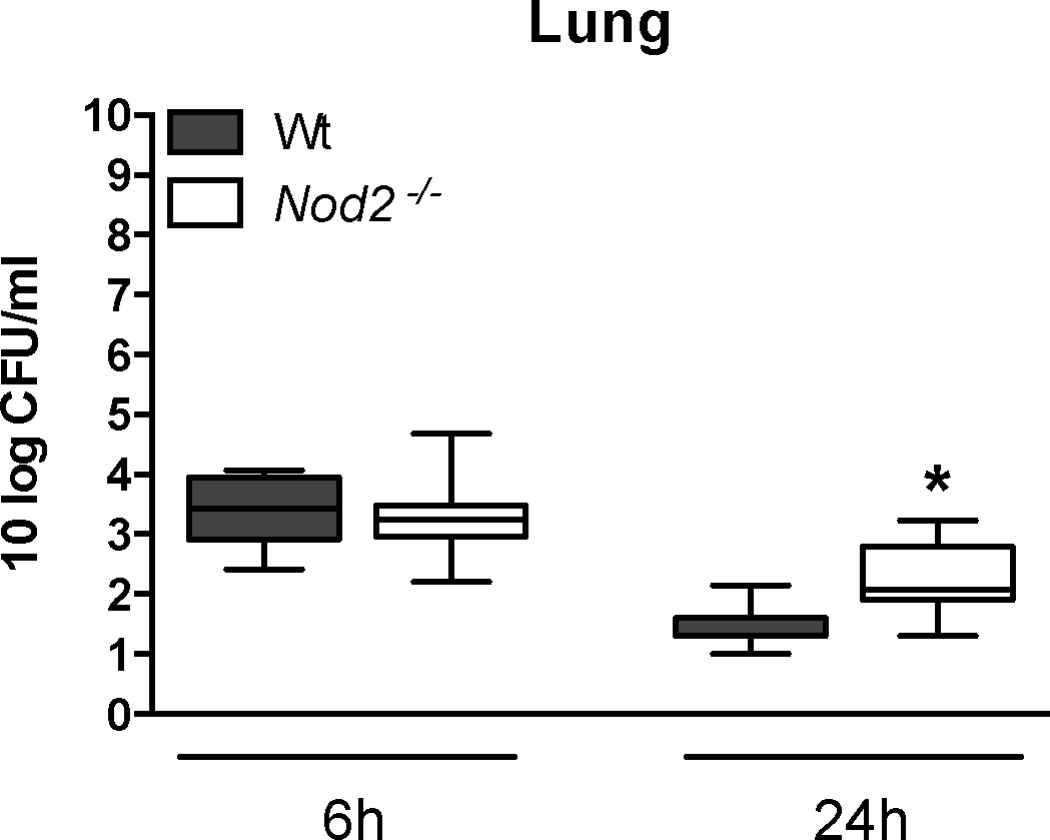
NOD2 deficiency results in defective pulmonary clearance of non-encapsulated mutant *S*. *pneumoniae* D39Δ*cps*. Wild-type (Wt) and *Nod2* ^*−/−*^ mice were intranasally infected with 10^8^ CFU of *S*. *pneumoniae* D39Δ*cps* and sacrificed 6 or 24 hours later. Bacterial loads were determined in lung homogenates. Data are expressed as box-and-whisker diagrams depicting the smallest observation, lower quartile, median, upper quartile and largest observation (8 mice per group at each time point); * *P*<0.05.

**Fig 6:**
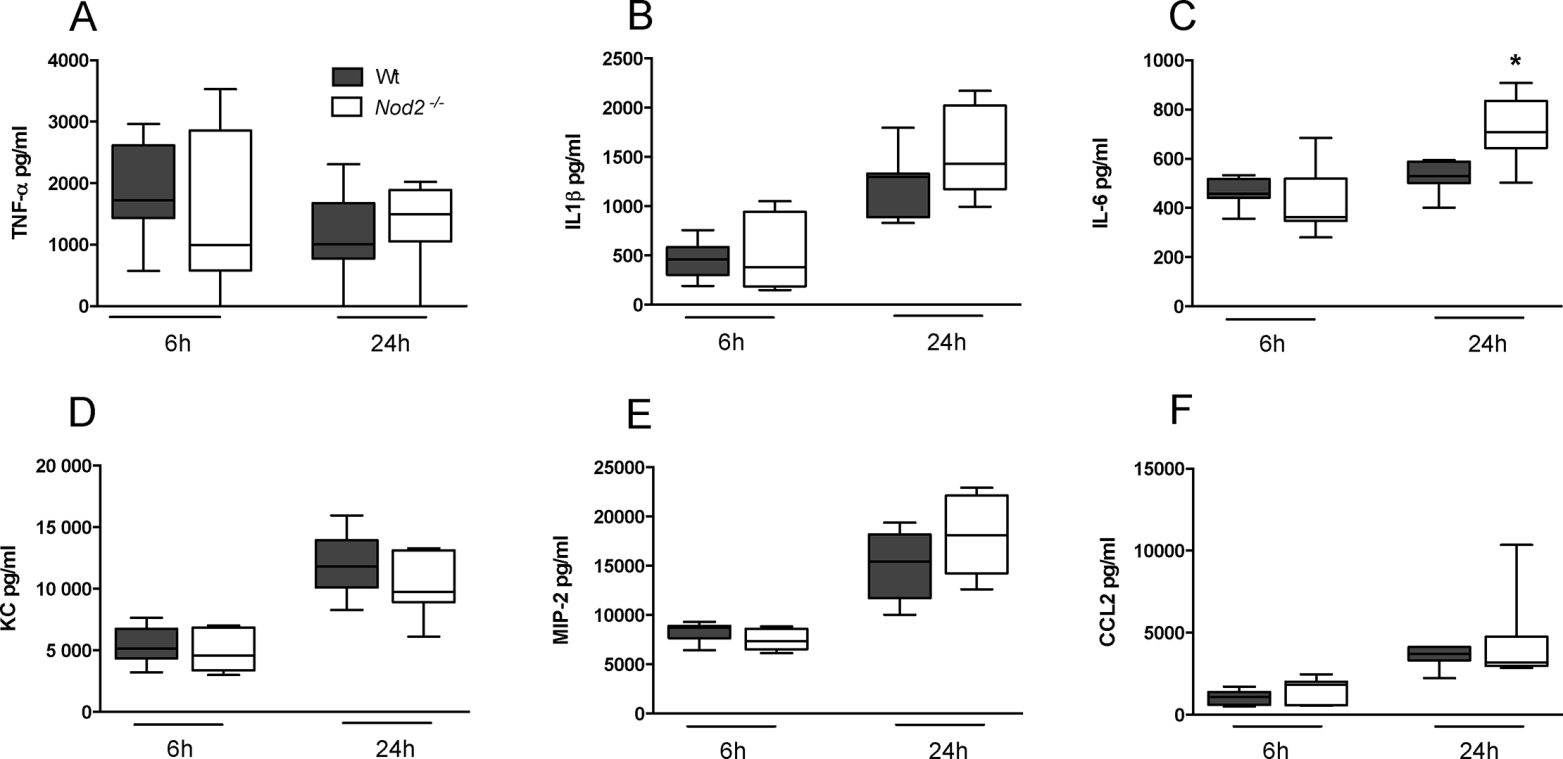
Cytokine and chemokine concentrations in lung homogenates of wild-type (Wt) and *Nod2* ^*-/-*^ mice during pneumococcal pneumonia caused by an unencapsulated mutant strain serotype 2 *S*. *pneumoniae* D39Δ*cps*. Proinflammatory cytokine (TNF-α, IL-1β, IL-6) and chemokine (KC, MIP-2 and CCL2) levels in lung homogenates at 6 and 24 hours after intranasal *S*. *pneumoniae* D39Δ*cps* infection in wild-type (Wt) and *Nod2* ^*-/-*^ mice. Data are expressed as box-and-whisker diagrams depicting the smallest observation, lower quartile, median, upper quartile and largest observation (8 mice per group at each time point); * *P*<0.05.

**Fig 7:**
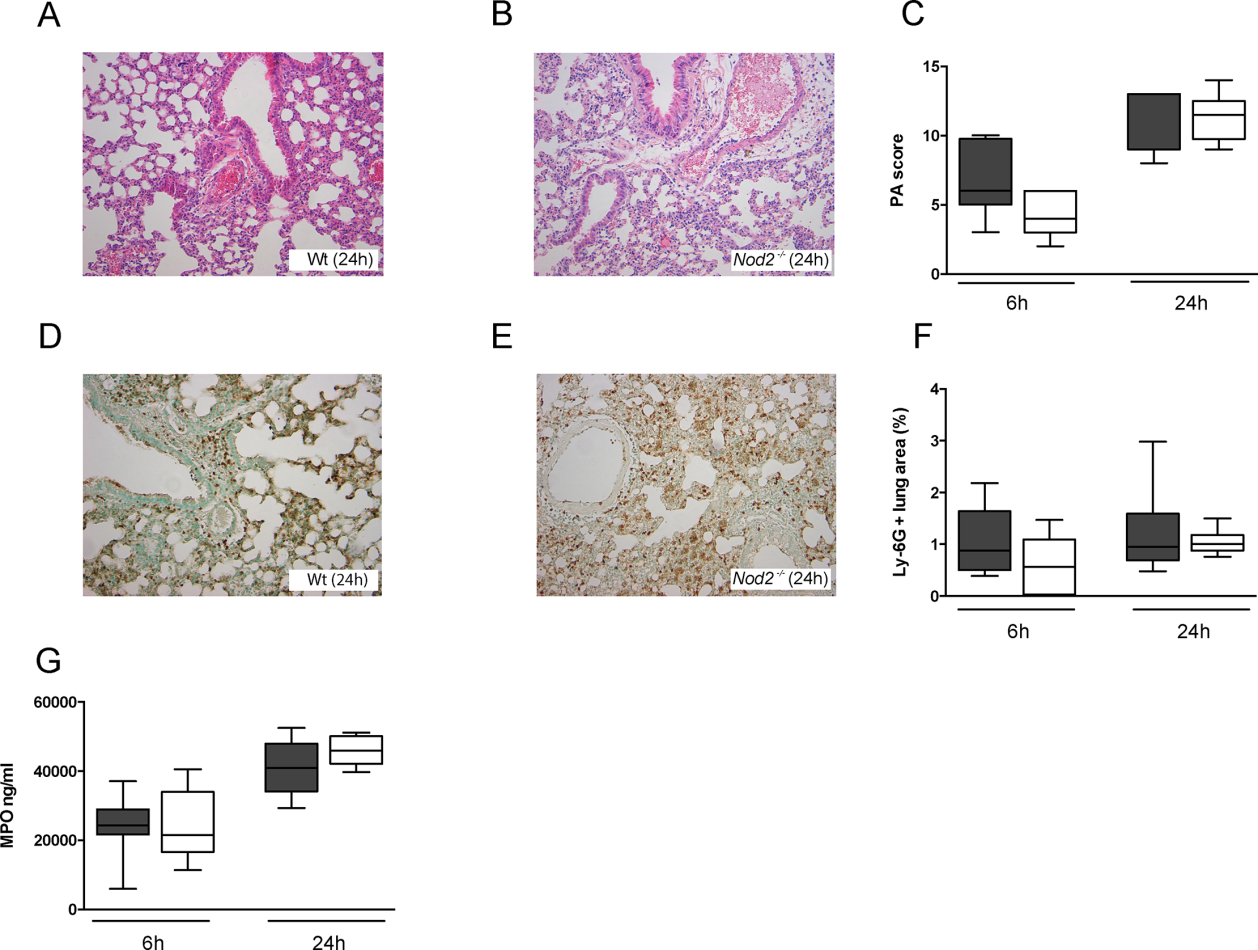
NOD2 deficiency does not influence lung pathology and neutrophil recruitment during pneumonia caused by an unencapsulated mutant *S*. *pneumoniae* D39Δ*cps*. Wild-type (Wt) and *Nod2* ^*−/−*^ mice were intranasally infected with 10^8^ CFU of *S*. *pneumoniae* D39Δ*cps* and sacrificed 6 or 24 hours later. Representative hematoxylin and eosin (HE) stainings of lung tissue of Wt (A) and *Nod2* ^*-/-*^ mice (B) 24 hours after inoculation with *S*. *pneumoniae* (original magnification ×200). Quantification of pulmonary Ly-6G positivity (F) and MPO levels in whole lung homogenates (G) 6 or 24 hours after intranasal infection with *S*. *pneumoniae* D39Δ*cps* of wild-type (Wt) and *Nod2* ^*−/−*^ mice. Representative neutrophil stainings (brown) of Wt (D) and *Nod2* ^*−/−*^ mice (E) 24 hours after induction of pneumococcal pneumonia are shown (original magnification ×200). Data are expressed as box-and-whisker diagrams depicting the smallest observation, lower quartile, median, upper quartile and largest observation (8 mice per group at each time point). Differences between groups were not significant.

We have recently shown that the thick polysaccharide capsule impairs recognition of Toll-like receptor (TLR) ligands expressed by *S*. *pneumoniae* [12]. To test whether this also holds true for NOD2, we next infected *Nod2* ^*-/-*^and Wt mice with the non-encapsulated mutant serotype 2 *S*. *pneumonia* D39Δ*cps*. Interestingly, we observed increased bacterial loads in lungs of *Nod2* ^*-/-*^ mice compared to Wt mice after 24 hours of infection (Fig 5; *P*<0.05). No dissemination into blood or distant organs was found in either Wt or *Nod2* ^*-/-*^ mice. Cytokine and chemokine levels in whole lung homogenates were not different between *Nod2* ^*-/-*^ and Wt mice with the exception of IL-6 concentrations, which were higher in lungs of *Nod2* ^*-/-*^ mice 24 hours after infection (Fig 6C; *P*<0.05). Likewise, histopathology scores (Fig 7A–7C), the number of Ly6 positive cells in lung tissue slides (Fig 7D–7F) and whole lung MPO concentrations (Fig 7G) were similar in *Nod2* ^*-/-*^ and Wt mice 6 and 24 hours after inoculation with *S*. *pneumoniae* D39Δ*cps*.

### NOD2 does not contribute to the host response during pneumonia caused by serotype 3 *S*. *pneumoniae*

**Fig 8:**
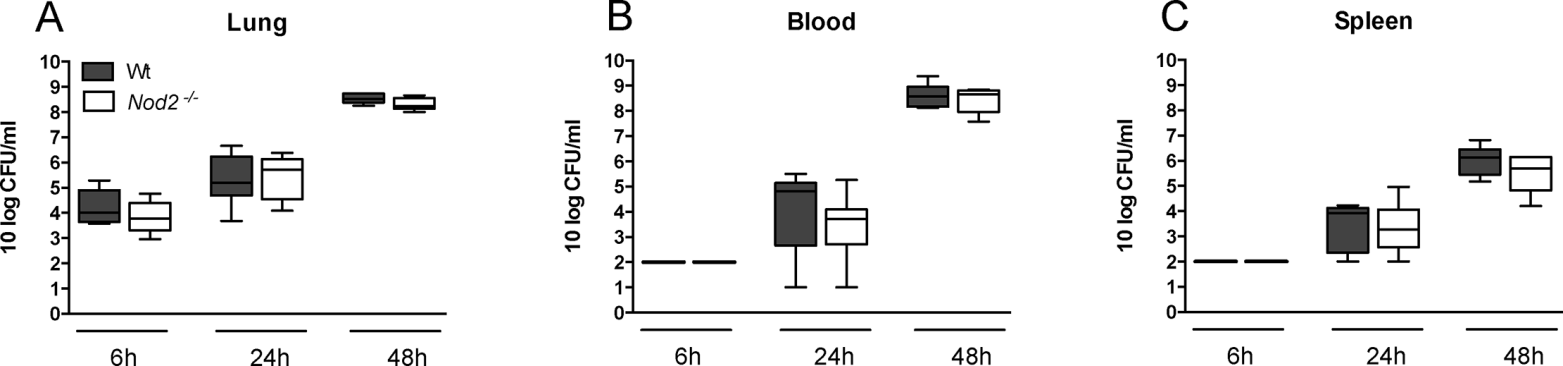
NOD2 deficiency does not impact on bacterial growth or dissemination during pneumonia caused by serotype 3 *S*. *pneumoniae*. Wild-type (Wt) and *Nod2* ^*−/−*^ mice were intranasally infected with 10^4^ CFU of *S*. *pneumoniae* (6303) and sacrificed 6, 24 or 48 hours later. Bacterial loads were determined in lung homogenates (A), blood (B) and spleen (C). Data are expressed as box-and-whisker diagrams depicting the smallest observation, lower quartile, median, upper quartile and largest observation (8 mice per group at each time point). Differences between groups were not significant.

In patients serotype 3 pneumococci are associated with severe disease [15–18]. Thus, to validate our findings of the limited role of NOD2 in host defense during pneumococcal pneumonia, we performed additional studies with the highly virulent serotype 3 *S*. *pneumoniae* 6303 strain. Similarly to experiments with the serotype 2 *S*. *pneumoniae* D39 strain, *Nod2* ^*-/-*^ and Wt mice had similar bacterial loads in lungs, blood and spleen at 6, 24 and 48 hours after infection (Fig 8A–8C).

## Discussion

NOD2 is a prominent member of the NLR family able to recognize microbial derived ligands in the cytosol [2]. While previous studies found a role for NOD2 in host defense against gram-negative pneumonia [19,20], limited data exists on the involvement of NOD2 in host defense in gram-positive pneumonia. In the present study we aimed to characterize the *in vivo* relevance of NOD2 in pneumococcal pneumonia. Since *S*. *pneumoniae* is sensed by NOD2 [6], we hypothesized that NOD2 deficiency would result in impaired innate immunity during lower airway infection by this common respiratory pathogen. However, we here found no role for NOD2 in host defense during pneumonia caused by two *S*. *pneumoniae* strains (serotype 2 D39 and the more virulent serotype 3 6303), as reflected by similar bacterial loads at the primary site of infection and distant organs in *Nod2* ^*-/-*^ and Wt mice at multiple time points after infection. Interestingly, when we used a mutant strain of *S*. *pneumoniae* lacking the thick polysaccharide capsule, we observed a modestly impaired pneumococcal clearance locally in lungs of *Nod2* ^*-/-*^ mice. Together these data suggest that NOD2, while contributing to antibacterial defense against normally avirulent pneumococci, does not play a significant role in the innate immune response during pneumococcal pneumonia *in vivo*.

To evaluate a functional role for NOD2 in pneumococcal pneumonia, we first assessed the capacity of neutrophils and alveolar macrophages, the main cell types involved in pulmonary clearance of *S*. *pneumoniae* [4,21], to internalize this pathogen *in vitro*. In accordance with an earlier report showing decreased uptake of *S*. *aureus* by bone marrow derived neutrophils [14], neutrophils and alveolar macrophages displayed impaired phagocytosis of *S*. *pneumoniae*. The mechanism by which NOD2 contributes to phagocytosis of *S*. *pneumoniae* might involve induction of matrix metalloproteinase (MMP)-9, considering that the pneumococcus induces MMP9 in a NOD2 dependent manner [7] and that MMP9 is important for phagocytosis of this bacterium by neutrophils [22]. A role for NOD2 in phagocytosis is not uniform: NOD2 does not contribute to internalization of *Mycobacterium* (*M*.) *bovis* BCG or *M*. *tuberculosis* by alveolar macrophages [23]. Similarly, bone marrow derived macrophages deficient for RIP2 –the adapter molecule for NOD2 –showed no defect in internalization of *Chlamydia pneumophila* [20].

In a previous study *Nod2* ^*-/-*^ mice showed reduced lung inflammation and neutrophil recruitment during *S*. *aureus* pneumonia [24]. Since *S*. *aureus* is rapidly cleared from the lungs of normal immune competent mice, this model used very high infectious doses (10^9^ CFU) [24], lessening its clinical relevance. As a consequence, these earlier results are difficult to compare with the current investigation, which not only involved another pathogen, but also much lower infectious doses of more virulent bacterial strains. Of the two pneumococcal strains tested, *S*. *pneumoniae* 6303 clearly is the most virulent. Indeed, while *S*. *pneumoniae* D39 is slowly cleared from mouse lungs, low dose infection with *S*. *pneumoniae* 6303 results in a high mortality in immune competent mice caused by a gradual growth and subsequent dissemination of bacteria [12,25,26]. Interestingly, despite the reduced capacity of NOD2 deficient macrophages and neutrophils to internalize *S*. *pneumoniae in vitro*, NOD2 did not contribute to either clearance of *S*. *pneumoniae* D39 or limiting the growth of *S*. *pneumoniae* 6303. Apparently the *in vitro* defect in phagocytosis of these cells is not relevant *in vivo* and is compensated for by other cells and/or mechanisms. Our results are in line with a recent paper showing that NOD2 deficiency does not impact on the clearance of *S*. *pneumoniae* from the upper airways in a model of nasopharyngeal colonization [8].

Lung inflammation was not altered in *Nod2* ^*-/-*^ mice during *S*. *pneumoniae* pneumonia, except for elevated levels of IL-6 and MIP-2 in whole lung homogenates. These results are in line with a previous report of elevated cytokine levels in lung homogenates of *Nod2* ^*-/-*^ mice during pneumonia caused by *Legionella* despite unaltered pulmonary bacterial loads [19]. Since pulmonary bacterial loads were slightly enhanced at 48 hours in NOD2 deficient mice, this might be an explanation for the higher IL-6 and MIP-2 levels observed. Alternatively, these data suggest NOD2 might reduce the proinflammatory response to *S*. *pneumoniae*. Since NOD2 is able to inhibit TLR2 mediated cytokine responses through NFκB [27], and TLR2 is implicated in the early inflammatory response to *S*. *pneumoniae* [26], one could speculate that the elevated IL-6 and MIP-2 levels in *Nod2* ^*-/-*^ mice are caused by absence of this inhibiting effect of NOD2 on TLR2 signalling. On the other hand, bone marrow derived macrophages lacking NOD2 produced less IL-6 *in vitro* compared to Wt cells when incubated with serotype 4 *S*. *pneumoniae* [8]. Considering the unaltered bacterial loads and lung pathology in *Nod2* ^*-/-*^ mice the modest effect of NOD2 on *S*. *pneumoniae* induced cytokine production is of little biological significance.

Interestingly, when we used an unencapsulated pneumococcal strain we found *Nod2* ^*-/-*^ mice to have increased pulmonary bacterial loads 24 hours after infection. We [12,13] and others [28,29] have previously shown a role for the pneumococcal capsule in the capacity of *S*. *pneumoniae* to multiply in the lower airways and to induce severe pneumonia. The polysaccharide capsule is a crucial virulence factor protecting *S*. *pneumoniae* from various harmful cellular processes including phagocytosis [30,31]. Since phagocytosis of *S*. *pneumoniae* is important for its degradation by lysozyme, releasing various ligands in the cytosol where it can be sensed by NOD2 [8], we hypothesize that in the absence of the capsule *S*. *pneumoniae* is delivered more easily to the cytosol where it can be sensed by NOD2. Recently, our group demonstrated that part of the virulence of encapsulated pneumococci relies on the capacity of the capsule to impair recognition of TLR ligands expressed by *S*. *pneumoniae* [12]. The present data suggest that the same mechanism may be at play for recognition of the pneumococcus by NOD2.

In conclusion, we here found no evidence for an important role for NOD2 in host defense during pneumonia caused by two different *S*. *pneumoniae* strains. Possibly, the innate immune response to *S*. *pneumoniae* is triggered by a simultaneous action of different PRRs. Indeed, during a pneumococcal colonization model, *Nod2* ^*-/-*^ mice were not hampered in clearance of *S*. *pneumoniae* from the upper airways; however, when mice lacking both NOD2 and TLR2 were used, impaired clearance of pneumococci was observed [8]. These data, together with findings that the bacterial capsule can shield *S*. *pneumoniae* from recognition by certain PRRs, exemplify the complex nature of immune defense against this common and clinically relevant pathogen.
